# *A Singular Case of* Hydrocephalus

**Published:** 1805-07-01

**Authors:** James S. Stringham

**Affiliations:** Professor of Chemistry in Columbia College, America


### *A singular Case of* HYDROCEPHALUS, *by* JAMES S. STRINGHAM, *M. D. Professor of Chemistry in Columbia College, America.*

THE subject of the following case is a female, who, when about nine days old, was attacked with symptoms of catarrh, in consequence of a sudden exposure to cold in the month of January, attended with considerable discharge from the mucous membrane of the nose, which continued till she was three months old, when it suddenly and entirely disappeared. About a week from this time, symptoms of hydrocephalus, accompanied with an enlargement of the head, commenced. Dr. Foot, of this city, was called upon, shortly after, to visit the child. The usual remedies, calomel, digitalis, epispastics, &c. were employed, but without any effect, the enlargement continuing tinuing to progress. An operation was, at length, thought advisable, with a view to ascertain whether any collection of fluid could be discovered between the dura or pia mater and the brain; but no such accumulation could be detected.

From the constant and gradual increase of the size of the head, every hope of recovery was abandoned, and her physician relinquished his attendance.

When I was requested to visit her, she was two years and three months old; consequently had laboured two years under this disease of the head, which I found of so astonishing a bulk, that I have been induced to present you not only with an account of its dimensions, but also with such other information, relative to the subject, as I have been able to procure from the mother of the child.

The distance between the internal angles of the eyes measured two inches; between the external extremities of the arches of the eye-brows, six and a quarter inches; between the upper extremities of the ears (measured directly across the parietal bones) thirteen and a half inches; between the superior part of the nasal and lamdoidal sutures, along the junction of the parietal bones, twenty inches; the circumference of the head was two feet six and a half inches.

It appears, from the mother's account, that, since the commencement of this enlargement of the head, a total suppression of the mucous discharge from the nose took place, which has never since been observed in the smallest degree, notwithstanding the exhibition of powerful errhines, which were incapable of producing even the slightest act of sneezing. The child cut her teeth at the usual period; but it is a circumstance worthy of remark, that, in a short time after, the upper row gradually softened and broke off even with the gums, while the under teeth remained firm, and are now perfectly sound. The pupils of the eyes are somewhat dilated, though a very evident contraction takes place in consequence of a sudden exposure to light. She hears uncommonly well, and can not only distinguish, with accuracy, the taste between different kinds of food, but, in this respect, exhibits much caprice. At present she prefers bread and milk to any other species of aliment. Her appetite is good, though by no means voracious. During the whole course of the disease, the alvine and urinary discharges have been perfectly regular and natural: her memory is, in no respect, impaired: she evinces a predilection for music; and such is the strength of of her recollection, that, after hearing an air once sung, she can repeat it, though a lapse of twenty-four hours should intervene. Sometimes she is very lively, and not unfrequently displays the hilarity of her spirits by laughing so loud as to be heard in every part of the room. She moves her left arm with apparent ease, though she cannot elevate the hand to her head. Over the voluntary muscles of the right arm she appears to have lost all controul. In damp weather the pain in her head is most frequent and violent. She is not much inclined to sleep; but, when in this state, moans constantly, and is frequently observed to startle and wake as if in a fright. In bed she is extremely restless, perpetually turning from the one side to the other. The motion of her legs is somewhat impaired. In every respect (the head excepted) her body is of a natural size, though, contrary to every thing that might be expected, she is rather taller than children in general of her age, and no marks of emaciation, or deficiency of action in the lymphatic system, are to be observed. The mother remarked to me, that her other children began to articulate many words so as to be understood when they were one year old; but that this child had not spoken any thing intelligibly till she had attained her second year; but so rapid was the progress she then made, that in three months, from that time, she could not only distinguish, by name, every object which surrounded her, but could communicate to her attendants, without any difficulty, those ideas with which her mind was most strongly impressed. The enlargement of this child's head still continues to progress; and, as she retains her strength, appetite, and spirits, she may probably still live a considerable time: hence it is impossible to say what may be the particular boundaries destined to limit its extension.

It is evident, from the very uncommon size of the head in this case, either that the bones of the cranium must be separated from each other, by the pressure of a fluid internally collected, or that the integuments must have undergone this astonishing dilation, in consequence of an accumulation of water between them and the bones of the head.

That the collection was internal and deep-seated, at the time of Dr. Foot's attendance, is evident, from the result of the operation already noticed.

That the bones composing the head of a child, at this age, should, by a mechanical force, be so separated, as to admit of an uncommon enlargement, I can easily conceive; but but that a quantity of fluid, sufficient to produce such an effect, should be accumulated in the ventricles of the brain, without being succeeded by marks of serious and more general derangement, was, I confess, altogether unexpected. None of those symptoms could be discovered, which are generally the effects of great pressure upon the nerves at their origin. The mucous membrane of the nose, indeed, appeared to be no longer irritable, and the motion of the arms and legs was somewhat impaired; but, in no other respect, was a diminution of nervous power indicated. Her hearing was uncommonly acute; her perception of objects perfectly distinct; the functions of the stomach and bowels were more regularly performed than is generally observed, even in a state of health; in short, the whole disease appeared to be confined to an increased size of the head, and a diminution of power over the voluntary muscles of the upper and lower extremities.

That the sagittal suture was opened, I have very little doubt, since, notwithstanding considerable pressure, no resistance similar to that of a bony substance could be perceived, although this was perfectly distinct in many other parts of the head.

Do not the circumstances which preceded this child's attack strongly corroborate the opinion of Dr. Garnett, that hydrocephalus is, in its first stage, an inflammatory affection? We find that no symptoms of the disease appeared till the increased discharge (which had been kept up, nearly three months, from the child's nose, in consequence of catarrh) was suddenly arrested. This preternatural secretion of the mucous membrane clearly evinced a determination of blood to the vessels of the head; and the discharge from the nose was the mean employed by Nature to obviate the effects of congestion. This once checked, the consequences soon became apparent, viz. a great turgidity of the vessels of the head, and an extravasation into the ventricles of the brain. Such is the plethoric state, particularly of the veins, in this part, that they are distended to more than double their usual size, and bear no small resemblance to those which have been injected for anatomical purposes.

Is it not probable, that if the suppressed discharge could have been immediately restored, or a vicarious one instituted on the first appearance of the hydrocephalic symptoms, all the horrors of the disease might, in this case, have been avoided? This circumstance affords an useful hint to practitioners, as to the *modus medendi* of hydrocephalus cephalus cephalus in general, and serves to point out in what way this affection may very frequently be induced.

The reduction of the upper teeth, from a bony to a soft and gelatinous consistence, may be easily accounted for, as the effect of an excess of phosphoric acid in the blood; but that the under row should not have been operated upon by the same cause, is a circumstance by no means to be so easily explained.

As the child is still living, I shall, for the present, suspend any further observations, and shall give every attention to the different phenomena which may take place from this time to her death, which, it is scarcely necessary to say, I consider as inevitable. These shall constitute the matter of a subsequent communication.

